# Effect of Electron Beam Irradiation on the Physicochemical and Structural Properties of Brown Rice Protein Isolates

**DOI:** 10.3390/foods15213875

**Published:** 2026-10-29

**Authors:** Dichen Ying, Junhao Li, Zhanchang Hu, Tianyu Qian, Yulong Bao, Zhiyang Yu, Zhen Yang

**Affiliations:** 1 Key Laboratory of Nuclear Agricultural Sciences of MARA of PRC, Institute of Nuclear Agricultural Sciences, Zhejiang University, Hangzhou 310058, China; 2 School of Food and Biological Engineering, Jiangsu University, Zhenjiang 212013, China

**Keywords:** electron beam irradiation, brown rice, brown rice protein isolate, protein conformation

## Abstract

Electron beam irradiation (EBI) is used to treat cereals, but its effects on isolated proteins recovered after irradiation of intact paddy rice remain unclear. EBI of rice before protein extraction may affect the properties of the recovered protein fraction. Paddy rice from Zhongzao 39 (ZZ39) and Jia 67 (J67) was exposed to 0–20 kGy EBI before dehulling and alkaline protein extraction. Brown rice color and moisture were measured, and the resulting brown rice protein isolates (BRPIs) were characterized using physicochemical, microscopic, spectroscopic, and thermal methods. Grain color varied across treatments, whereas moisture values remained within a narrow range for each rice material. Aqueous protein solubility at pH 7.0 was low and generally decreased after irradiation. Hydrodynamic diameter and surface hydrophobicity showed patterns with variations across the tested dose levels, while the major reducing SDS-PAGE band positions were retained. Fitted amide I profiles, fluorescence intensities, and thermal-transition parameters also differed among treatments. These observations characterize proteins recovered after grain irradiation and subsequent extraction, precipitation, and freeze-drying. The two rice materials showed different descriptive response patterns. Overall, the findings identify changes that should be considered when recovering proteins from irradiated rice. EBI altered BRPI association and conformation without detectable shifts in surface potential or principal reducible subunit distribution. These findings supported the potential of EBI as a physical treatment for modulating brown-rice protein properties, provided that irradiation conditions are selected according to cultivar and the target quality attribute.

## 1. Introduction

Brown rice is obtained by removing the hull while retaining the bran, germ, and endosperm. Compared with polished rice, it contains more dietary fiber, lipids, proteins, minerals, vitamins, and phenolic compounds [1]. However, the retained bran and germ also make brown rice more susceptible to oxidative deterioration, discoloration, and other storage-related quality losses [2]. These changes can reduce sensory quality and market value. It is therefore important to determine how postharvest treatments affect both the visible quality of brown rice and the properties of its recoverable protein fraction. Rice protein is nutritionally valuable and has relatively low allergenic potential, but its broad use as a food ingredient is limited by poor aqueous dispersibility and compact, glutelin-rich storage assemblies [3,4]. Its technological behavior depends on hydration, surface charge, hydrophobic interactions, molecular flexibility, and protein association [5,6]. These factors also influence the ability of plant proteins to stabilize food interfaces [7]. Structural modification is consequently pursued to improve rice-protein dispersibility and processing performance without compromising nutritional quality.

Plant-protein modification can be achieved through chemical, enzymatic, and physical routes. Major physical approaches include high-pressure processing, ultrasound, homogenization or microfluidization, pulsed electric fields, thermal treatment, and irradiation [8,9]. Their effects depend on treatment intensity and the initial protein matrix, and excessive processing may promote oxidation or renewed association [10,11]. Research on non-thermal processing therefore emphasizes the need to define an effective treatment range [12,13]. Recent studies show that rice responses to cold plasma depend on treatment conditions and target quality attributes. Air- and argon-based dielectric barrier discharge cold plasma (DBD-CP) differently affected enzyme activity, starch properties, and volatile profiles in lightly milled rice [14], whereas nitrogen-vacuum cold-plasma pretreatment produced condition-dependent effects on subsequent modification of rice starch with octenyl succinic anhydride (OSA) [15]. These findings underscore the need to optimize non-thermal treatments for specific quality objectives. For rice protein, pH shifting combined with ultrasound and heat increased solubility from approximately 2.7% to 91.8% and produced less compact, more dispersed aggregates [16]. Industry-scale microfluidization combined with pH cycling increased dispersibility from approximately 1.4% to 78.3% through aggregate dissociation and conformational rearrangement [17]. Because both protocols included pH adjustment, they represent physicochemically conditioned physical treatments rather than purely physical modification. EBI offers a distinct non-thermal route because its electron source can be switched on and off, treatment is rapid, and no radioisotope source is required. Appropriately controlled EBI can reduce microbial and pest loads and extend food shelf-life, although quality responses remain dependent on dose and food composition [18].

Previous studies have examined EBI effects on either intact cereal grains or isolated proteins. In oat and barley, 10 MeV EBI altered lipid stability during storage [19]. In brown rice, 1–5 kGy EBI caused little immediate change in moisture content, while low-dose treatments inhibited lipase activity and microbial growth and slowed increases in fatty acid value and yellowness during storage [20]. In inoculated husked rice, 7.5 kGy electron-beam irradiation reduced Bacillus cereus to below 10 CFU/g, illustrating that microbial inactivation depends on the organism and treatment conditions [21]. This published example provides context for irradiation intensity rather than a sterilization target for the present protein-focused study. At the protein level, enzymolysis-assisted EBI altered rice-protein structure and antioxidant activity [22], whereas EBI of isolated rice protein changed aggregation, conformation, and functional properties [23]. Chickpea and oat/barley protein isolates showed dose-dependent changes in solubility, particle size, hydrophobic exposure, and conformation, with response maxima at different doses [24,25]. Related studies on soybean 11S globulin and okara protein reported intermediate-dose changes in solubility or interfacial performance accompanied by structural rearrangement [26,27]. Walnut-meal and pea proteins also exhibited changes in conformation and functionality [28,29]. However, whole-grain studies have primarily addressed storage quality, whereas protein studies have generally irradiated proteins after isolation. It remains unclear how EBI applied to paddy rice affects brown rice quality and the physicochemical and multiscale structural properties of the subsequently isolated proteins.

The present study examined brown rice quality and the physicochemical and structural properties of BRPIs recovered from two paddy rice cultivars irradiated at 0–20 kGy. Zhongzao 39 (ZZ39) is a medium-maturing early-season indica cultivar developed in Zhejiang Province, whereas Jia 67 (J67) is a conventional late-season japonica cultivar used for single-season production. Treating paddy rice places irradiation at a grain-processing stage and permits grain quality and recovered protein properties to be examined within the same processing sequence. It evaluates the outcome of irradiation followed by extraction, rather than the superiority of either processing order. This design provides an experimental basis for selecting EBI conditions that account for both brown rice quality and protein properties.

## 2. Materials and Methods

### 2.1. Materials

Paddy rice of the early-season indica cultivar ZZ39 and the late-season japonica J67 were purchased from Zhejiang Wuwangnong Seed Industry Co., Ltd. (Hangzhou, China). The potassium (silver) dichromate dosimeter (ZJTC-200928-L) was purchased from Zhongjin Irradiation Co., Ltd. (Shenzhen, China). The reagents, including urea, glycine, ethylenediaminetetraacetic acid (EDTA), 5,5′-dithiobis(2-nitrobenzoic acid) (DTNB), and tris(hydroxymethyl)aminomethane (Tris) were purchased from Aladdin Biochemical Technology Co., Ltd. (Shanghai, China). The 8-Anilino-1-naphthalenesulfonic acid (ANS) was obtained from Yuanye Bio-Technology Co., Ltd. (Shanghai, China). Sodium dodecyl sulfate (SDS), Coomassie Brilliant Blue G-250, and methanol were supplied by Sinopharm Chemical Reagent Co., Ltd. (Beijing, China). All other reagents were of analytical grade, and ultrapure water was used for solution preparation unless otherwise stated.

### 2.2. Experimental Design and Analytical Methods

#### 2.2.1. Electron Beam Irradiation and Brown Rice Protein Isolation

The EBI process was performed using the method reported by Tang et al. with slight modifications [19]. Approximately 1 kg of paddy rice from each cultivar was spread uniformly in a resealable plastic bag to form a layer less than 1 cm thick, and the potassium (silver) dichromate dosimeters were placed beneath the rice sample at the bottom of the supporting tray to measure the actual irradiation doses. The irradiation experimental unit was the paddy-rice batch for each cultivar and target dose. One batch per cultivar was irradiated at target doses of 1, 5, 10, 15, or 20 kGy using a 10 MeV high-energy electron linear accelerator (DZ-10/20, China National Nuclear Corporation, Beijing, China), operated at a beam power of 15 kW and an average beam current of 1.5 mA. The accelerator had a scanning width of 80 cm, and the reported dose uniformity across the scanning range was ≤±5%. The distance from the titanium window to the stainless-steel tray (80 cm × 60 cm) was 70 cm. Untreated samples served as the 0 kGy controls. The nonirradiated dosimeter was used as the blank for spectrophotometric measurements (GENESYS 30, Thermo Scientific, Waltham, MA, USA). The corresponding absorbed doses were 1.08, 5.06, 10.40, 14.92, and 19.60 kGy, respectively. After irradiation, the paddy rice was dehulled to obtain brown rice, which was milled, passed through a 60-mesh sieve, and stored at −20 °C. The moisture and ash contents of the nonirradiated ZZ39 control brown rice flour were 11.28 ± 0.01% and 1.12 ± 0.21%, respectively, and the values for the nonirradiated J67 control were 13.32 ± 0.15% and 1.17 ± 0.30%, respectively. The BRPI recovery values for ZZ39 at 0, 1, 5, 10, 15, and 20 kGy were 6.68 ± 0.19%, 7.45 ± 0.24%, 6.67 ± 0.16%, 7.13 ± 0.38%, 7.58 ± 0.21%, and 6.45 ± 0.29%, respectively. The corresponding values for J67 were 6.02 ± 0.28%, 5.64 ± 0.11%, 6.40 ± 0.23%, 5.91 ± 0.17%, 5.58 ± 0.24%, and 5.73 ± 0.34%, respectively. The determinations were performed using three independent BRPIs.

BRPI was isolated by alkaline extraction and isoelectric precipitation using a procedure adapted from de Souza et al. [30]. Brown rice flour (50 g) was dispersed in 750 mL of 0.18% NaOH and stirred at 30 °C for 30 min. After centrifugation at 3380× *g* for 5 min, the supernatant was collected. The residue was washed once with 250 mL of 0.18% NaOH, and the wash was combined with the first supernatant. The combined supernatant was adjusted to pH 4.8 with 0.1 M HCl to precipitate the protein. *T*_o_ minimize variability introduced by extraction and drying, all control and irradiated samples were processed under the same pH, temperature, centrifugation, neutralization, freezing, and drying conditions [31]. The precipitate was recovered by centrifugation, neutralized to pH 7.0 with 0.1 M NaOH, frozen at −80 °C for 24 h, and freeze-dried at −55 °C and <20 Pa for 48 h.

#### 2.2.2. Color Measurement

The brown rice color was measured using a portable spectrophotometer (MiniScan EZ 4500, HunterLab, Reston, VA, USA). Before each measurement, the instrument was calibrated using the black and white standards supplied by the manufacturer. Brown rice was spread uniformly in a transparent glass Petri dish at a depth sufficient to cover the measurement aperture completely and minimize light leakage and background interference. Three independent measurements were obtained for each sample. For each cultivar, total color difference (*ΔE*) was calculated from the mean color coordinates of each irradiated treatment relative to its nonirradiated control using Equation (1). Because *ΔE* was calculated from treatment-mean coordinates, it was treated as a descriptive index and was not subjected to inferential analysis:

(1) $$\Delta E\;=\;\sqrt{\left[{\left(L^{*}\;-\;L_{0}^{*}\right)}^{2}+\;{\left(a^{*}\;-a_{0}^{*}\;\right)}^{2}+\;{\left(b^{*}\;-\;b_{0}^{*}\right)}^{2}\right]}$$

where *L*^∗^, *a*^∗^, and *b*^∗^ denote the color coordinates of an irradiated treatment, and *L*_0_^∗^, *a*_0_^∗^, and *b*_0_^∗^ denote those of the corresponding 0 kGy control.

#### 2.2.3. Moisture Content Determination

Moisture content was determined using a HE83/02 halogen moisture analyzer (Mettler-Toledo Instruments Co., Ltd., Greifensee, Switzerland) according to the AOAC method for cereal moisture [32]. Approximately 2.0 g of brown rice flour was distributed uniformly on an aluminum pan and dried at 105 °C in standard mode. The measurement ended automatically when the mass-loss rate fell below 1 mg per 50 s. Each sample was analyzed independently in triplicate.

#### 2.2.4. Solubility Determination

The BRPI (50 mg) was dispersed in 5 mL of ultrapure water, adjusted to pH 7.0, and stirred magnetically for 2 h at room temperature. The dispersion was centrifuged at 6000× *g* and 25 °C for 10 min. Protein concentration in the supernatant was determined using the Coomassie Brilliant Blue assay at 595 nm. Before sample analysis, a standard curve was prepared using BSA solutions of different concentrations [26]. Soluble protein (% of BRPI mass) was calculated using Equation (2), with the result expressed as the mass of soluble protein relative to the total BRPI powder mass:

(2) $$\mathrm{Solubility}\;=\;\frac{m_{1}}{m_{0}}\times \;100\%$$

where *m*_1_ is the mass of protein recovered in the supernatant, and *m*_0_ is the total mass of BRPIs powder added to the assay (50 mg). Three independent determinations were performed.

#### 2.2.5. Zeta Potential and Particle Size Measurement

Average hydrodynamic diameter and particle-size distribution were measured by dynamic light scattering (DLS), and zeta potential was measured by electrophoretic light scattering using a Nano ZS90 analyzer (Malvern, Malvern, UK). BRPI was dispersed at 1 mg/mL in 10 mM phosphate buffer (pH 7.0) and equilibrated in the instrument for 2 min at 25 °C. The dispersant was defined as an aqueous phase. For each sample, three independent measurements were performed, each comprising 15 automatically acquired instrument runs.

#### 2.2.6. Scanning Electron Microscopy Analysis

The dry-state morphology of BRPI was observed using field-emission scanning electron microscopy (SEM; SU-8010, Hitachi, Tokyo, Japan) according to Li et al. [25]. A small amount of each powder was mounted on conductive adhesive and sputter-coated with gold before observation at an accelerating voltage of 3.0 kV. Micrographs were acquired at 500× magnification, and each image includes a 100 μm scale bar.

#### 2.2.7. Surface Hydrophobicity Determination

Surface hydrophobicity (*H*_0_) was determined using 8-anilino-1-naphthalenesulfonic acid (ANS) as a fluorescent probe. BRPI was prepared at 1 mg/mL in 10 mM phosphate buffer (pH 7.0) and diluted to 0.05–0.25 mg/mL. Each dilution (4 mL) was mixed with 20 μL of 8 mM ANS and incubated in the dark for 15 min. Fluorescence intensity was measured at excitation and emission wavelengths of 390 and 470 nm, respectively, using slit widths of 5 nm. *H*_0_ was defined as the slope obtained by linear regression of fluorescence intensity against protein concentration. Each treatment was analyzed independently in triplicate.

#### 2.2.8. Determination of Sulfhydryl and Calculated Apparent Disulfide Index

Free sulfhydryl (SH_F_), total sulfhydryl (SH_T_), and a calculated apparent disulfide index (S–S) were evaluated using a DTNB assay adapted from Zhang et al. [23]. BRPI was prepared at 1 mg/mL in 10 mM phosphate buffer (pH 7.0). SH_T_ was measured in Tris–glycine–EDTA–SDS buffer (pH 8.0) containing 8 M urea, whereas SH_F_ was measured in the same buffer without urea. After reaction with DTNB for 30 min, absorbance was measured at 412 nm. SH_F_ and SH_T_ contents and the apparent S–S index were calculated using Equations (3)–(5), respectively:

(3) $${\mathrm{SH}}_{F\;}=\;\frac{73.53\;\times \;A_{1\;}\times \;D}{C}$$

(4) $${\mathrm{SH}}_{T}=\frac{73.53\;\times {\;A}_{2}\;\times \;D}{C}$$

(5) $$S–S=\frac{{\mathrm{SH}}_{T}-{\mathrm{SH}}_{F}}{2}$$

where *A*_1_ and *A*_2_ are the absorbance values used to calculate SH_F_ and SH_T_, respectively, *D* is the dilution factor, and *C* is the protein concentration (mg/mL). SH_F_ and SH_T_ are expressed as μmol/g protein. Three independent replicate determinations were performed.

#### 2.2.9. SDS-PAGE Analysis

Reducing SDS-PAGE was performed using a 4–20% precast polyacrylamide gel (Tiandirenhe, Zhongshan, China) and a Vazyme MP201 molecular-weight marker (12–180 kDa; Vazyme, Nanjing, China). Protein solution (20 μL, 5 mg/mL) was mixed with 5 μL of 5× loading buffer and heated at 95 °C for 5 min. The entire 25 μL mixture was loaded into each well. Electrophoresis was conducted at a constant 120 V for 75 min, until the tracking dye approached the bottom of the gel. The gels were stained with Coomassie Brilliant Blue G-250, destained for 2 h in methanol/glacial acetic acid/water (1:1:8, *v*/*v*/*v*), and photographed using a white-light transilluminator.

#### 2.2.10. Fourier Transform Infrared Spectroscopy Analysis

FTIR spectra were acquired using a Nicolet iS50 spectrometer (Thermo Scientific, Waltham, MA, USA). Protein powder was mixed with KBr at a mass ratio of 1:100, pressed into a pellet, and scanned 32 times over 4000–400 cm^−1^ at a resolution of 4 cm^−1^. The amide I region was interpreted using established FTIR approaches for estimating protein secondary structure [33]. The spectrum was baseline-corrected using OMNIC 8.0. The amide I band (1700–1600 cm^−1^) was deconvoluted and fitted with Gaussian components using PeakFit 4.12. The algorithms are 2nd Deriv Zero and Lin, used for Gaussian response function deconvolution and second derivative method; the reported fit used seven iterations and achieved *r*^2^ > 0.99. The relative peak areas were used to estimate the α-helix, β-sheet, β-turn, and random-coil proportions. The fitted amide-I components were assigned to β-sheet (1600–1640 cm^−1^), random coil (1640–1650 cm^−1^), α-helix (1650–1660 cm^−1^), and β-turn (1660–1700 cm^−1^).

#### 2.2.11. Intrinsic Fluorescence Spectroscopy

BRPI was dispersed at 0.5 mg/mL in 10 mM phosphate buffer (pH 7.0) and equilibrated for 1 h at room temperature. Intrinsic fluorescence spectra were recorded using an F-4500 fluorescence spectrophotometer (Hitachi, Tokyo, Japan) at an excitation wavelength of 280 nm. Emission spectra were collected from 300 to 450 nm at 0.2 nm intervals using excitation and emission slit widths of 5 nm and a scanning speed of 1200 nm/min. Each sample was measured in triplicate. The spectra were corrected for buffer/background, scattering, and inner-filter effects before interpretation.

#### 2.2.12. Differential Scanning Calorimetry

Thermal transitions were measured with a Q20 differential scanning calorimeter (DSC, TA Instruments, New Castle, DE, USA) following Li et al. [25]. Freeze-dried BRPI powder (2 mg) was weighed into an aluminum pan and hermetically sealed without added water. Samples were heated from 30 to 180 °C at 10 °C/min. The onset temperature (*T*_o_), peak transition temperature (*T*_d_), transition enthalpy (Δ*H*), and half-height peak width (*ΔT*_1/2_) were obtained using TA Universal Analysis software (version 4.5A, TA Instruments, USA). Each recovered sample was measured in analytical triplicate. The software normalized the integrated transition heat to the entered mass of the weighed BRPI powder. Accordingly, the enthalpy is expressed as J/g BRPI.

### 2.3. Statistical Analysis

Unless otherwise indicated, results are presented as mean ± standard deviation (SD) from three replicate determinations. Comparisons among the available dose batches within each cultivar were made by one-way analysis of variance using SPSS 26 (IBM SPSS Statistics, Armonk, NY, USA), followed by Tukey’s multiple-comparison test at *p* < 0.05. The replicate determinations characterize variation within the available treatment batches. Thus, *p*-values and significance letters describe comparisons conditional on the sampled batches. FTIR secondary-structure estimates and *ΔE* values were treated descriptively. No inferential comparisons were performed between cultivars. Figures and tables were prepared using Origin 2021 (OriginLab Corporation, Northampton, MA, USA) and GraphPad Prism 10.1.2 (GraphPad Software, Boston, MA, USA).

## 3. Results and Discussion

### 3.1. Effect of Electron Beam Irradiation on Brown Rice Color

**Figure 1:**
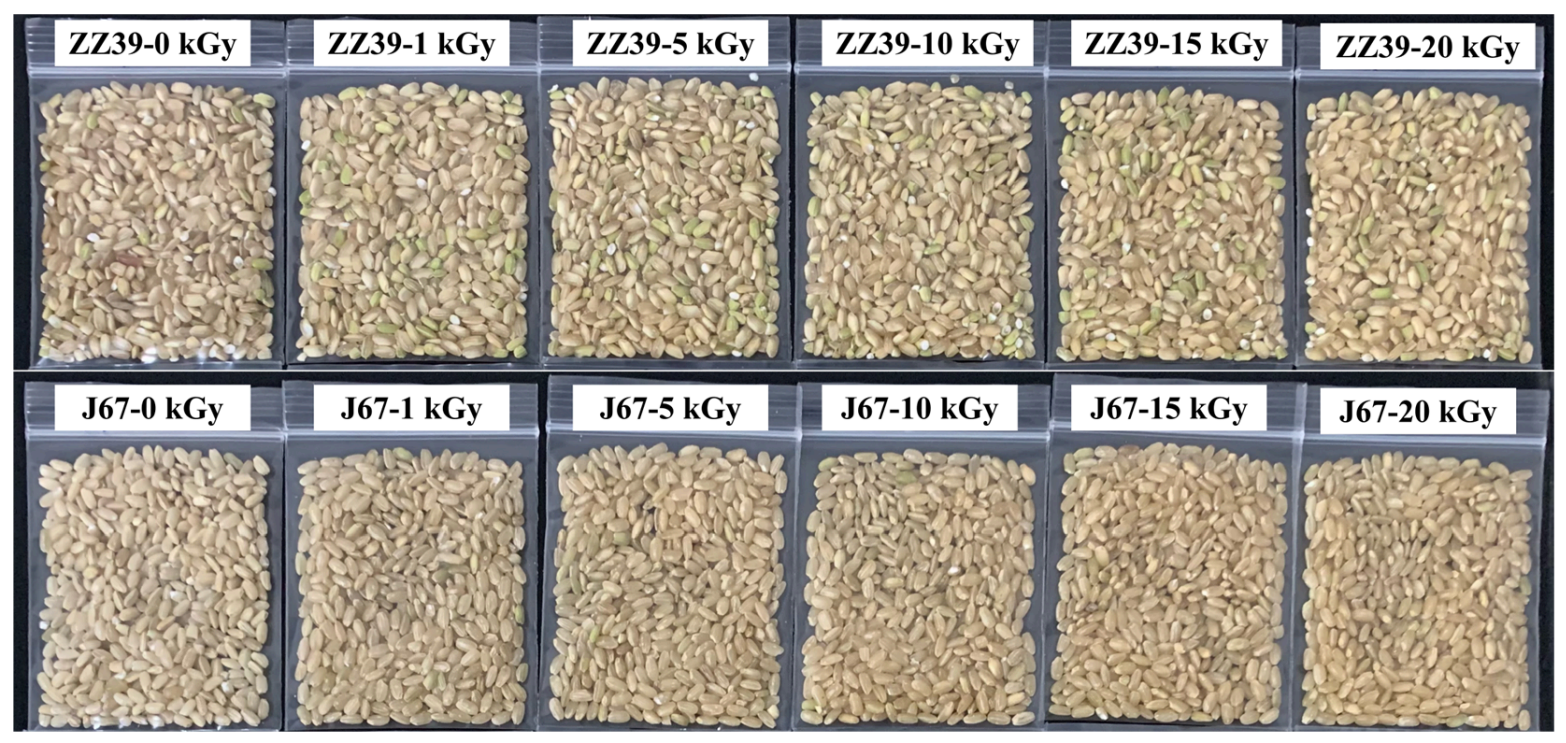
Appearance of ZZ39 and J67 brown rice treated by EBI at 0, 1, 5, 10, 15, and 20 kGy.

Color change was evaluated using the CIELAB coordinates *L*^∗^, *a*^∗^, and *b*^∗^, which represent lightness, red–green chromaticity, and yellow–blue chromaticity, respectively; *ΔE* represents the overall color difference from the corresponding nonirradiated control. Within the EBI treat batches, statistical comparisons indicated differences in at least one coordinate for both ZZ39 and J67 (Figure 1 and Table 1). In ZZ39, *L*^∗^ increased from 53.49 ± 0.82 to 56.08 ± 0.94 at 5 kGy and then decreased to 50.21 ± 0.63 at 20 kGy. The *a*^∗^ value was the lowest at 15 kGy, whereas *b*^∗^ did not differ significantly among doses. With the control (0 kGy) defined as 0.00, *ΔE* ranged from 0 to 3.32. In J67, all irradiated groups had lower *L*^∗^ values (42.81–44.66) than the control (48.70 ± 0.20), while *a*^∗^ and *b*^∗^ differed among doses; *ΔE* ranged from 0.00 to 5.90. Thus, the immediate color response of J67 was dominated by reduced lightness, whereas ZZ39 showed smaller overall deviations from its control sample. J67 showed a greater color change than ZZ39, mainly through reduced lightness.

Ren et al. reported that 1–5 kGy EBI had little immediate effect on *L*^∗^ and *a*^∗^ but slightly increased *b*^∗^ and the yellowness index of Zhengnong No. 1 brown rice [20]. This limited response was closer to the relatively small *ΔE* values observed for ZZ39, whereas the consistent decrease was not reported in that study. Other processing methods have been found to change the color of rice. For instance, cold plasma treatment increased the *L*^∗^ value of lightly milled rice from 62.93 to 76.69 [34], while superheated steam increased the value from 67.28 to 73.05 [35]. Protein changes may also accompany rice discoloration during storage. Liu et al. found that accelerated rice yellowing increased median protein-particle size and *H*_0_ and altered sulfhydryl/disulfide status [36]. Although storage-induced yellowing differs from the immediate EBI response examined here, this finding supports evaluating grain color together with protein properties. The present results therefore show that the immediate color response to EBI differed between ZZ39 and J67.

### 3.2. Effect of Electron Beam Irradiation on Brown Rice Flour Moisture Content

**Figure 2:**
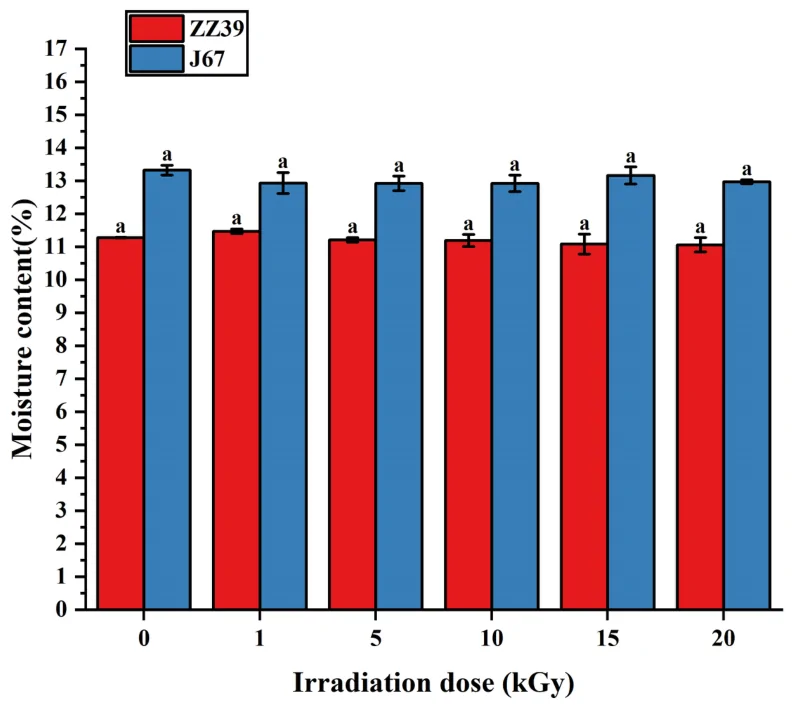
Moisture content of ZZ39 and J67 brown rice flour treated with different EBI doses (0–20 kGy). Values are presented as mean ± SD (*n* = 3). Identical lowercase letters indicate no significant differences among the available dose batches within the same rice material (*p* > 0.05).

Moisture content was measured to determine whether EBI changed the bulk water content of brown rice flour. Values remained within 11.06–11.47% for ZZ39 and 12.92–13.32% for J67, with no statistically significant differences among the available dose batches in either rice material (*p* > 0.05; Figure 2). Ren et al. found no significant difference in the immediate moisture content of brown rice treated with 0–5 kGy EBI (14.35–14.72%; *p* > 0.05) [20]. Thus, EBI treatment at 1–20 kGy did not greatly change the moisture content of brown rice.

### 3.3. Effect of Electron Beam Irradiation on Protein Solubility

**Figure 3:**
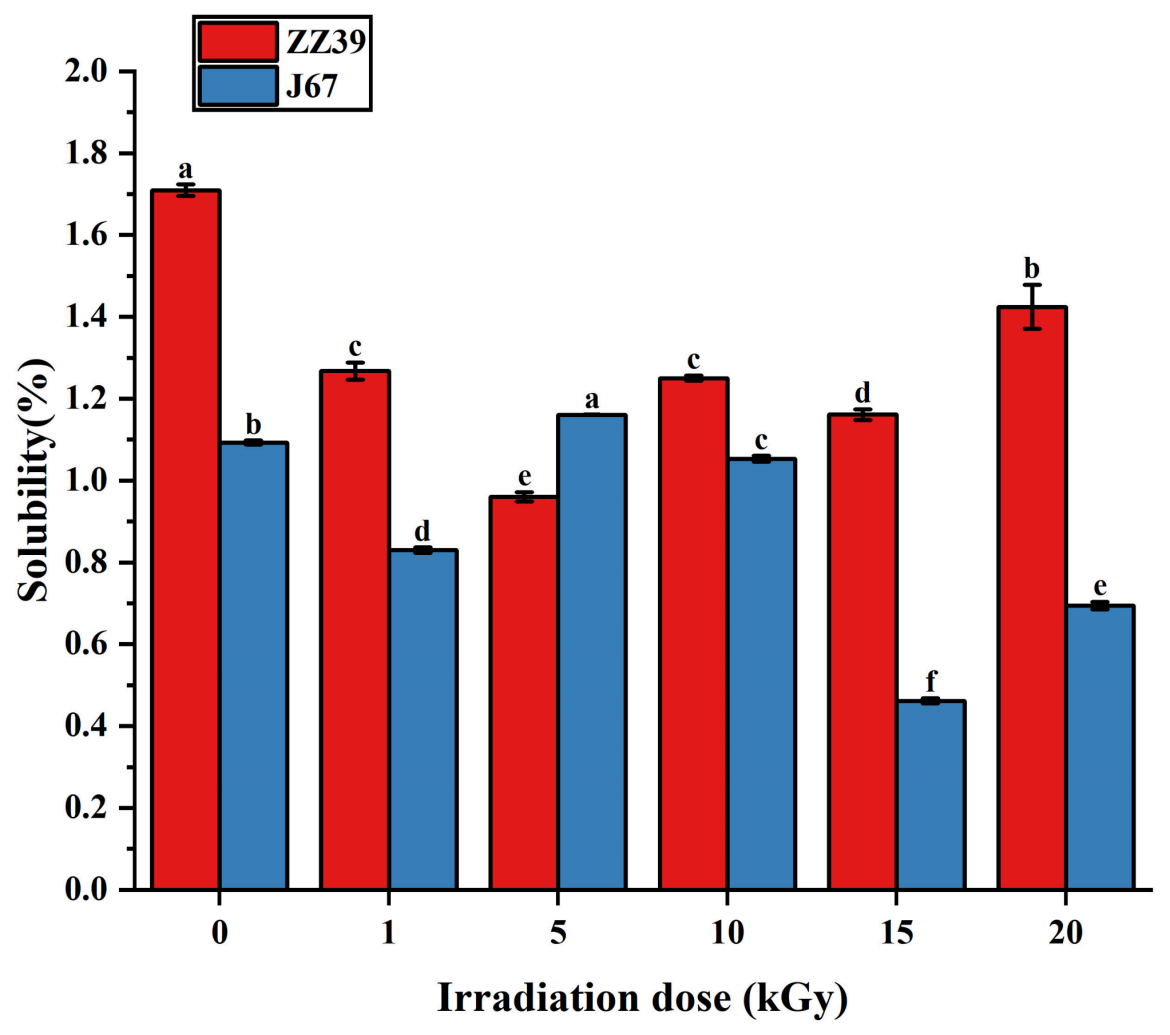
Solubility of ZZ39 and J67 BRPIs treated with EBI at 0–20 kGy. Values are presented as mean ± SD (*n* = 3). Different lowercase letters indicate significant differences among the available dose batches within the same rice material (*p* < 0.05).

Protein solubility describes protein recovery in the aqueous supernatant under specified extraction and assay conditions [6]. The ZZ39 and J67 isolates contained 73.32 ± 0.53% and 70.24 ± 0.48% crude protein, respectively. Statistical comparisons indicated differences in this quantity among the available treatment batches within each BRPI (Figure 3). For ZZ39, solubility decreased from 1.71% in the control to 1.27% at 1 kGy and 0.96% at 5 kGy, and then increased to 1.25%, 1.16%, and 1.42% at 10, 15, and 20 kGy, respectively. For J67, solubility decreased from 1.09% to 0.83% at 1 kGy, increased to 1.16% at 5 kGy, and then decreased to 1.05%, 0.46%, and 0.69% at 10, 15, and 20 kGy, respectively.

Li et al. reported that the solubility of oat and barley protein isolates increased to a maximum at 10 kGy and then decreased at higher EBI doses [25]. This increase followed by a decrease resembles the transient recovery observed for J67, although its maximum occurred at 5 kGy. In soybean 11S globulin, Bai et al. attributed the increase below 6 kGy to greater exposure of hydrophilic groups and the subsequent decrease to the formation of less soluble aggregates [26]. These mechanisms provide plausible explanations for the brief improvement in J67 and the lower solubility observed at higher doses, but differences among protein sources preclude direct comparison of absolute values.

Because ultrapure water without solubilizing additives was used as the dispersant, the measured values represent baseline aqueous dispersibility rather than the maximum solubility attainable after additional modification. Li et al. reported substantially higher rice-protein solubility when EBI was combined with enzymatic hydrolysis [37]; those values are not directly comparable with the present nonenzymatic assay.

### 3.4. Effect of Electron Beam Irradiation on Zeta Potential and Particle Size

**Figure 4:**
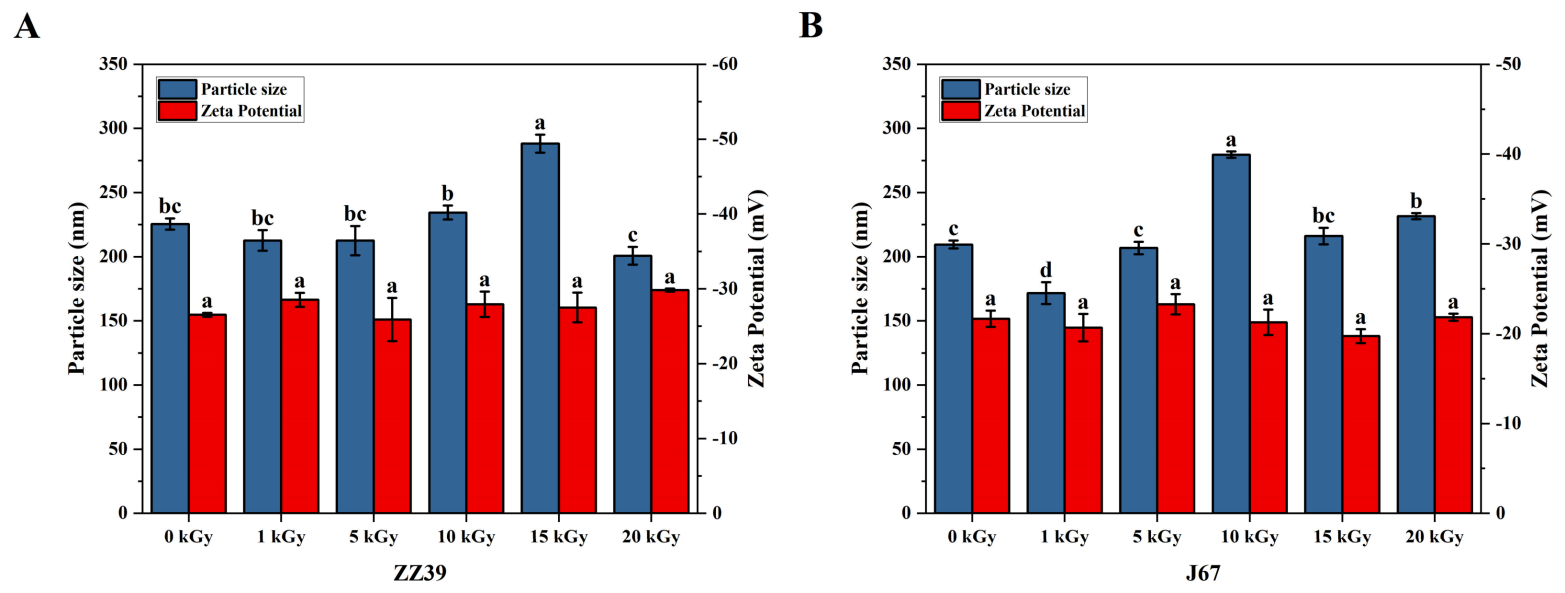
Zeta potential and average hydrodynamic diameter of (**A**) ZZ39 and (**B**) J67 BRPIs following EBI treatment with different doses. Values are presented as mean ± SD (*n* = 3). Different lowercase letters indicate significant differences among the available dose batches within the same rice cultivar *(p* < 0.05).

Zeta potential reflects the average electrophoretic surface potential of dispersed BRPI particles, whereas dynamic light scattering measures their hydrodynamic diameter. All BRPI dispersions were negatively charged at pH 7.0 (Figure 4). Zeta potential ranged from −25.90 to −29.83 mV for ZZ39 and from −19.73 to −23.27 mV for J67, with no statistical significant differences among the available dose batches in either cultivar. Therefore, EBI did not change the average surface potential under the assay conditions.

**Figure 5:**
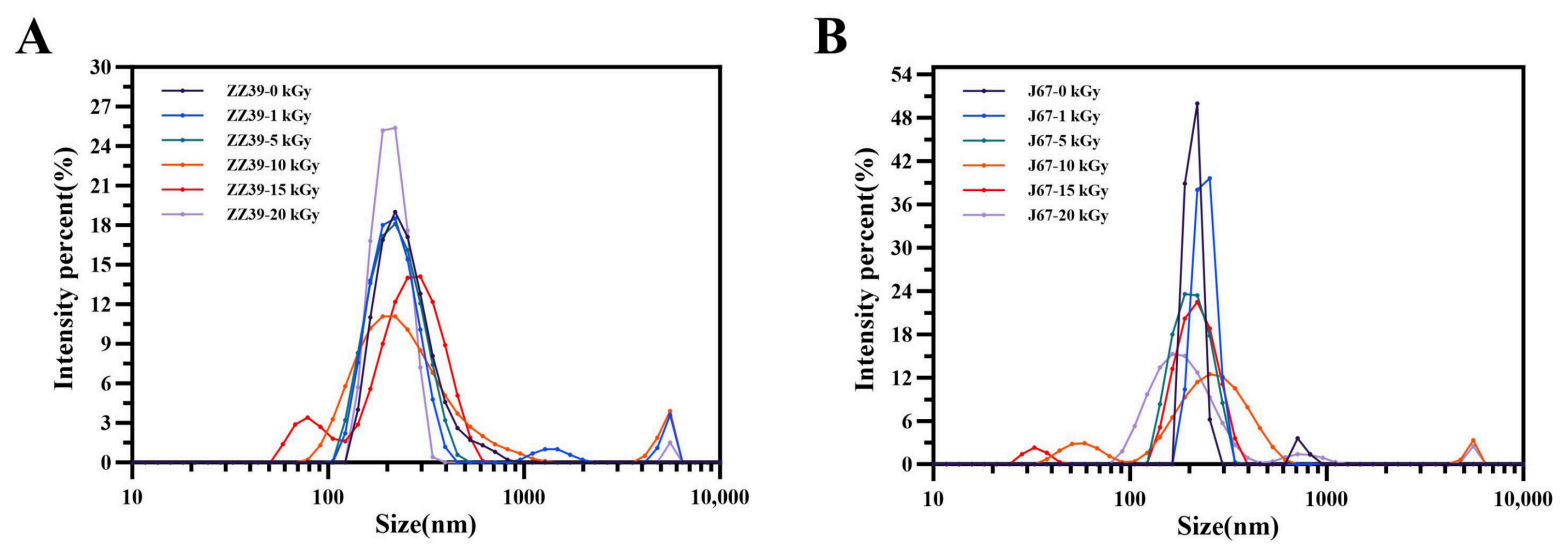
Particle-size distributions of (**A**) ZZ39 and (**B**) J67 BRPIs following treatment with different EBI doses.

As shown in Figure 4, the Z-average hydrodynamic diameter varied across irradiation doses in both cultivars; the dose at which the largest mean diameter was observed differed between ZZ39 and J67. ZZ39 had an initial diameter of 225.43 nm, decreased to approximately 212.6 nm at 1–5 kGy, then increased to a maximum of 288.13 nm at 15 kGy, and decreased to 200.77 nm at 20 kGy. J67 decreased from 209.53 nm in the control to 171.63 nm at 1 kGy, then increased to a maximum of 279.57 nm at 10 kGy, and declined to 216.10–231.63 nm at 15–20 kGy. The particle size values plotted in Figure 4 are the Z-average hydrodynamic diameters measured by DLS. Across the tested doses, polydispersity index (PDI) ranged from 0.349 to 0.782 for ZZ39, and from 0.384 to 0.889 for J67. The relatively high PDI values indicate heterogeneous dispersions, which may be caused by the aggregation of BRPIs that may also influence Z-average. Small secondary peaks at approximately 5–7 μm were also observed in the intensity-based particle-size distributions (Figure 5), suggesting a minor population of much larger particles in the dispersions. These peaks could reflect the association of protein particles into larger hydrodynamic aggregates rather than the enlargement of individual protein molecules. Electron beam irradiation has been reported to induce aggregation in oat and barley protein isolates at high doses [25], making particle association one possible explanation for the micron-scale peaks observed here.

Li et al. reported that the average particle sizes of oat and barley protein isolates initially decreased to minima at 10 and 5 kGy, respectively, and then increased at high EBI doses [25]. This initial decrease followed by enlargement was also observed in both BRPIs, although the largest particles occurred at 15 kGy for ZZ39 and 10 kGy for J67. Gamma-irradiated rice protein also showed a minimum particle size at an intermediate dose [38]. Thus, our results may indicate that particle-size change varied across the tested dose levels, and that the dose associated with particle enlargement depended on the protein material. In the BRPIs, the dose-associated changes in Z-average hydrodynamic diameter were not accompanied by a statistically significant change in zeta potential at pH 7.0 (Figure 4). Thus, variation in measured particle size did not necessarily coincide with a change in the mean electrophoretic surface potential under these conditions.

### 3.5. Effect of Electron Beam Irradiation on Apparent Morphology

**Figure 6:**
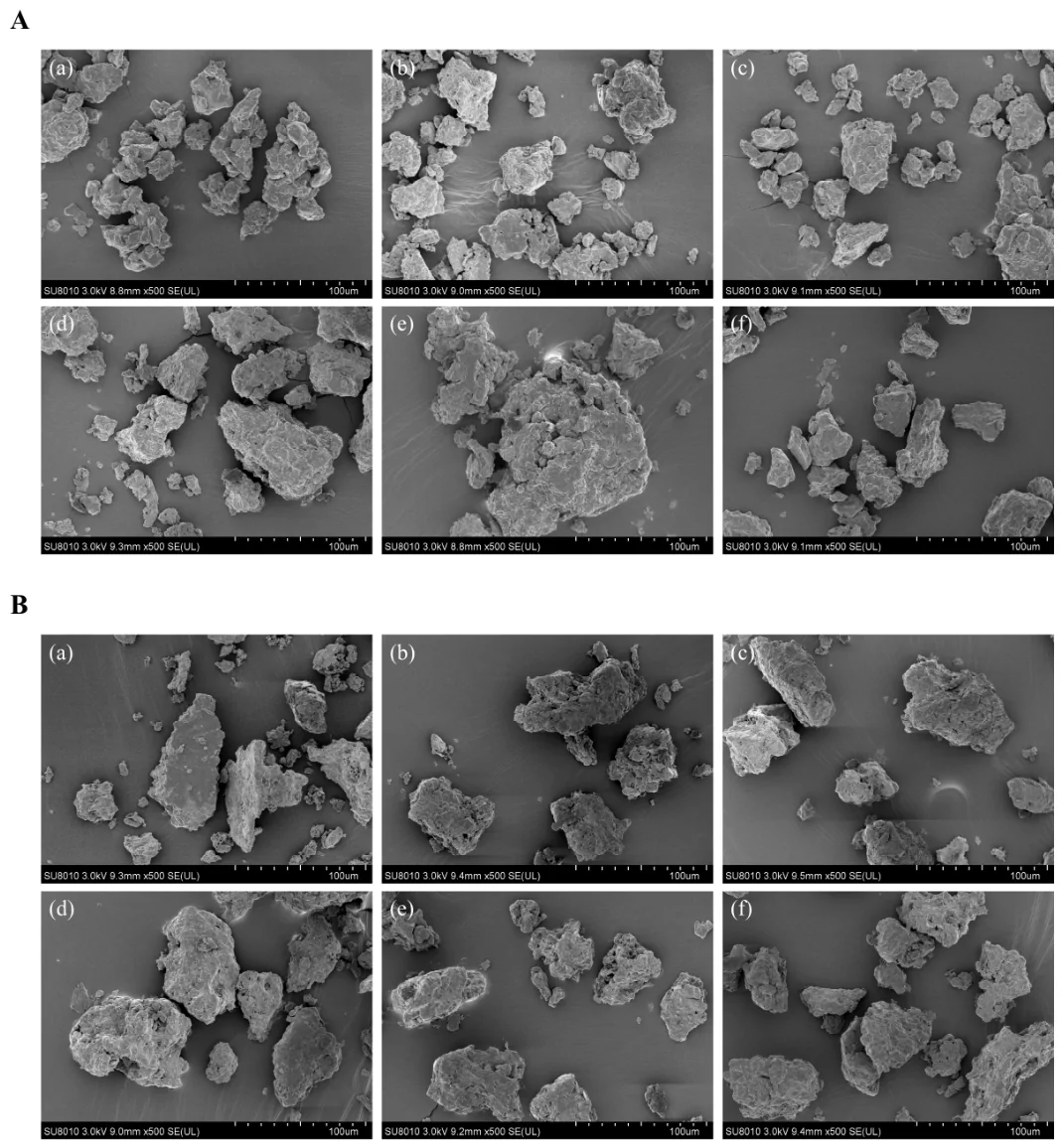
Scanning electron microscopy images of (**A**) ZZ39 and (**B**) J67 BRPIs following treatment with different EBI doses. In each panel, (**a**–**f**) correspond to 0, 1, 5, 10, 15, and 20 kGy, respectively. Images were acquired at 500× magnification; scale bars = 100 μm.

SEM was used to compare the dry-state morphology of BRPI powders after irradiation. The nonirradiated samples consisted of irregular plate-like particles and agglomerates with rough, folded surfaces (Figure 6). ZZ39 contained clusters assembled from smaller flakes, whereas J67 showed a more heterogeneous mixture of large plate-like particles and smaller fragments.

Compared with the nonirradiated ZZ39 sample, the 1 and 5 kGy samples contained smaller and more separated irregular particles, whereas larger, closely associated particles were observed at 10 kGy. Agglomeration was most pronounced at 15 kGy, while the 20 kGy sample again contained more discrete fragments (Figure 6A). For J67, particles remained relatively discrete at 1–5 kGy compared with the control. Larger and more closely associated particles were most apparent at 10 kGy, whereas the 15 and 20 kGy samples contained fewer contiguous agglomerates and a broader range of particle sizes (Figure 6B). The doses showing the most pronounced agglomeration by SEM corresponded to the maximum hydrodynamic diameters measured for ZZ39 and J67. Although SEM characterizes dry-state morphology, the SEM and DLS results provided observations of particle association in different sample states.

Zhang et al. reported that 5 kGy EBI disrupted compact rice-protein aggregates and increased the proportion of smaller particles, whereas 10–30 kGy produced additional cracks and fragments [23]. Li et al. similarly observed fragmentation of oat and barley protein particles at low-to-intermediate EBI doses, followed by the reappearance of larger aggregates at higher doses [25]. The smaller particles observed at low doses and the agglomeration observed at selected higher doses in the present BRPIs were consistent with these reported morphological responses. The strongest particle association was observed at different doses in ZZ39 and J67, while SEM and DLS provided qualitatively complementary observations in the dry and dispersed states, respectively.

### 3.6. Effect of Electron Beam Irradiation on Surface Hydrophobicity

**Figure 7:**
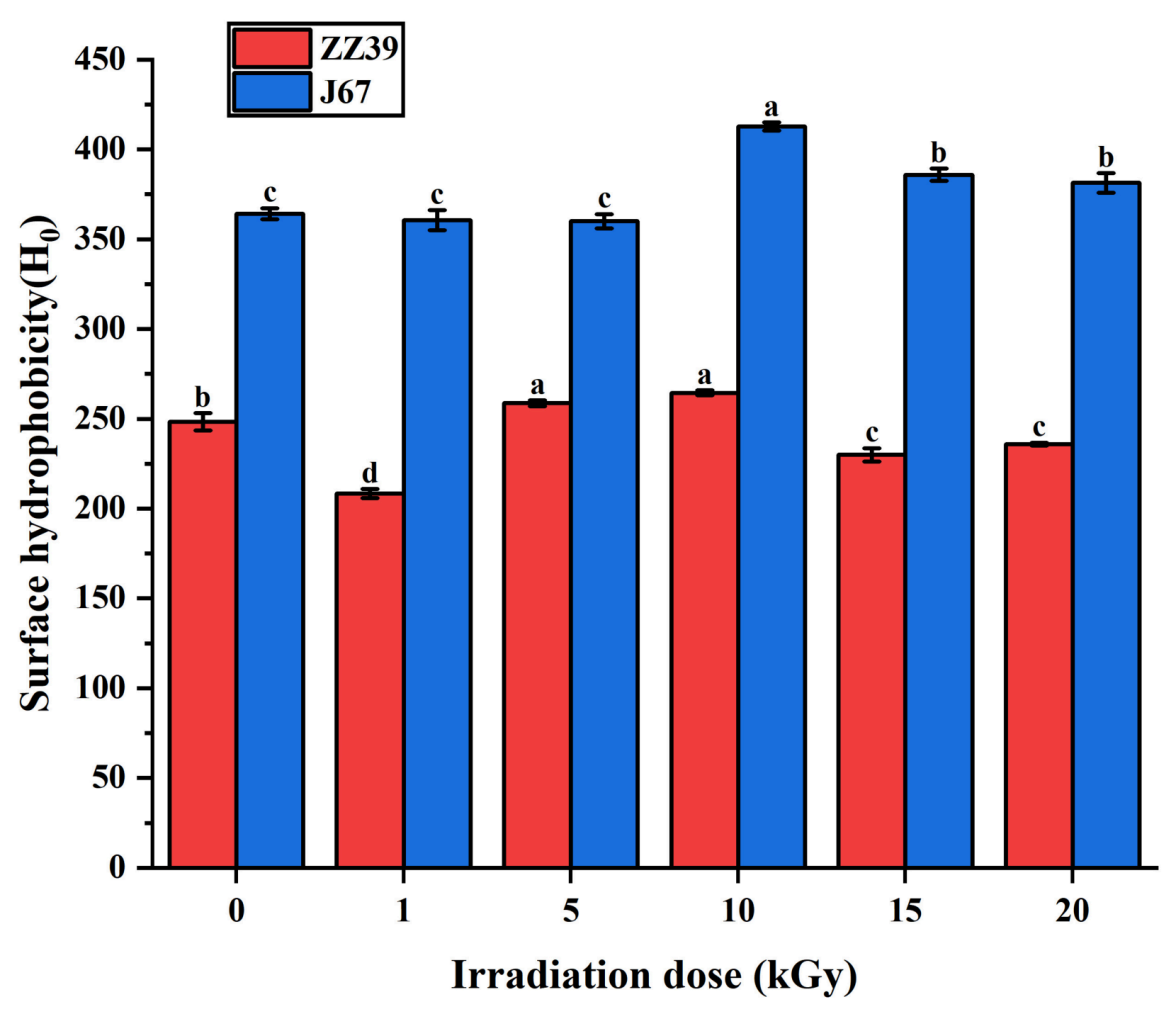
Surface hydrophobicity (*H*_0_) of ZZ39 and J67 BRPIs following treatment with different EBI doses. Values are presented as mean ± SD (*n* = 3). Different lowercase letters indicate significant differences among dose batches within the same rice material (*p* < 0.05).

Statistical comparisons indicated differences in surface hydrophobicity (*H*_0_) among the treatment batches of both BRPIs (Figure 7). For ZZ39, *H*_0_ decreased from 248.30 to 208.37 at 1 kGy, increased to 258.70 at 5 kGy and 264.43 at 10 kGy, and then declined to 229.94–235.90 at 15–20 kGy. J67 had an initial *H*_0_ of 364.13, remained statistically unchanged at 1–5 kGy, increased to 412.70 at 10 kGy, and then decreased while remaining above the control at 15–20 kGy. Both BRPIs therefore reached their highest *H*_0_ at 10 kGy.

Li et al. reported that *H*_0_ values of oat and barley protein isolates increased to maxima at 15 kGy and then declined at 20 kGy [25]. Chickpea protein showed a similar increase at 10–15 kGy followed by a decrease at 20 kGy [24], while goat-milk casein and chicken myofibrillar protein reached their highest *H*_0_ values within lower intermediate-dose ranges [39,40]. These results are consistent with the increase and subsequent decrease observed in both BRPIs, although the maximum occurred at different doses among protein materials.

*H*_0_ did not change in parallel with hydrodynamic diameter across all treatments. ZZ39 reached its largest diameter at 15 kGy, when *H*_0_ was below the control, whereas J67 reached maximum values for both indices at 10 kGy. Thus, *H*_0_ reflects the net exposure of hydrophobic regions and should not be interpreted as a direct measure of protein unfolding or particle size.

### 3.7. Effect of Electron Beam Irradiation on Sulfhydryl Contents and Calculated Apparent Disulfide Index

**Figure 8:**
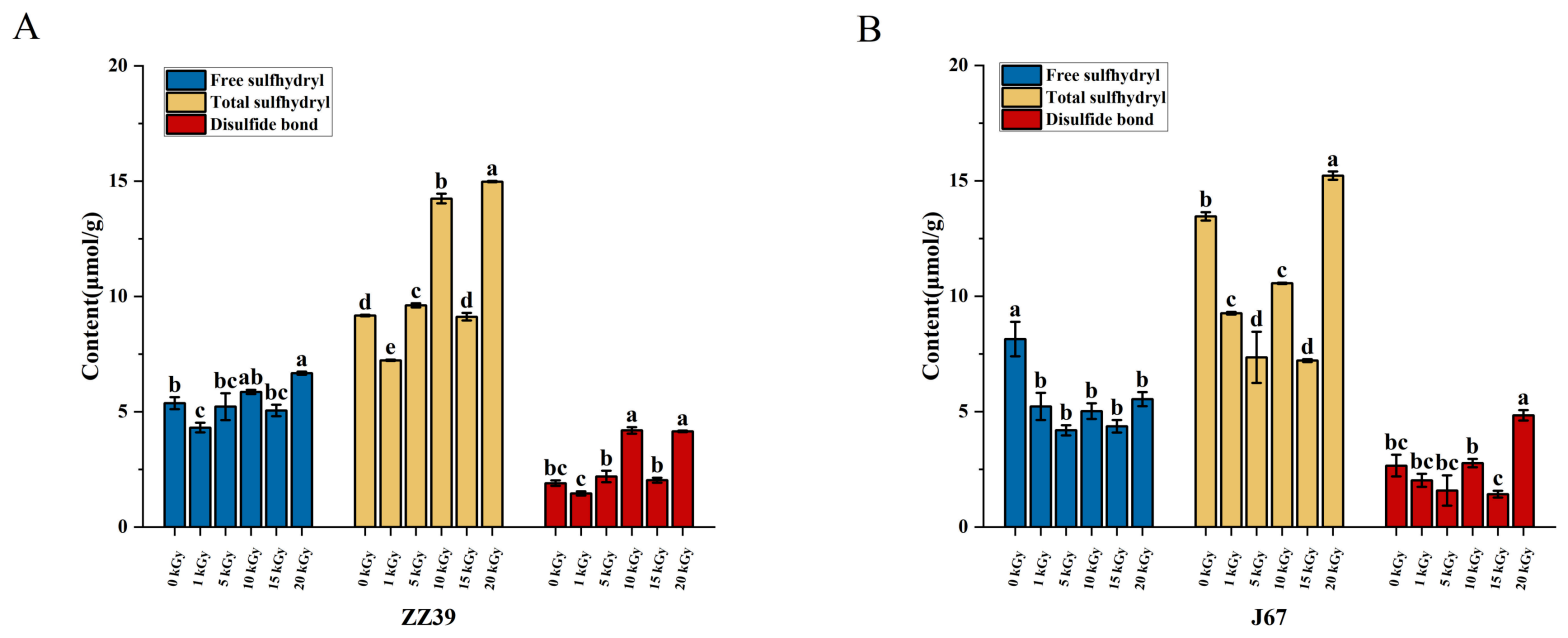
Free sulfhydryl (SH_F_), total sulfhydryl (SH_T_), and calculated apparent disulfide index (S–S) of (**A**) ZZ39 and (**B**) J67 BRPIs following treatment with different EBI doses. Values are presented as mean ± SD (*n* = 3). Different lowercase letters within each index indicate significant differences among the available dose batches of the same rice material (*p* < 0.05).

Free sulfhydryl (SH_F_), total sulfhydryl (SH_T_), and a calculated apparent disulfide index (S–S) were used to characterize changes in cysteine accessibility and the operational thiol/disulfide balance. In ZZ39, SH_F_ decreased from 5.37 to 4.31 μmol/g at 1 kGy, returned toward the control at 5–15 kGy, and increased to 6.67 μmol/g at 20 kGy (Figure 8). SH_T_ ranged from 7.23 to 14.98 μmol/g and was highest at 10 and 20 kGy, yielding calculated S–S maxima of 4.19 and 4.15 μmol/g, respectively. In J67, the control SH_F_ and SH_T_ values were 8.14 and 13.46 μmol/g. Both decreased at low-to-intermediate doses, whereas SH_T_ increased to 15.22 μmol/g at 20 kGy. The calculated apparent S–S index decreased to 1.42 μmol/g at 15 kGy and increased to 4.84 μmol/g at 20 kGy.

The present data support a dose-dependent redistribution of accessible and total thiol groups, with increased SH_T_ and calculated S–S at 20 kGy in both BRPIs but different SH_F_ responses. Irradiated oat and barley proteins also showed matrix-specific thiol responses. Oat-protein SH_F_ reached 23.90 ± 1.10 μmol/g at 15 kGy, whereas barley protein showed a marked high-dose increase in calculated S–S accompanied by lower SH_F_ [25]. Rice protein treated at up to 30 kGy showed increased exposed sulfhydryl content together with lower total sulfhydryl and higher S–S content [23]. These studies and the present results show that SH_F_, SH_T_, and calculated S–S can change in different directions because exposure, burial, and oxidation contribute simultaneously to the measured thiol balance.

### 3.8. Effect of Electron Beam Irradiation on SDS-PAGE Profiles

**Figure 9:**
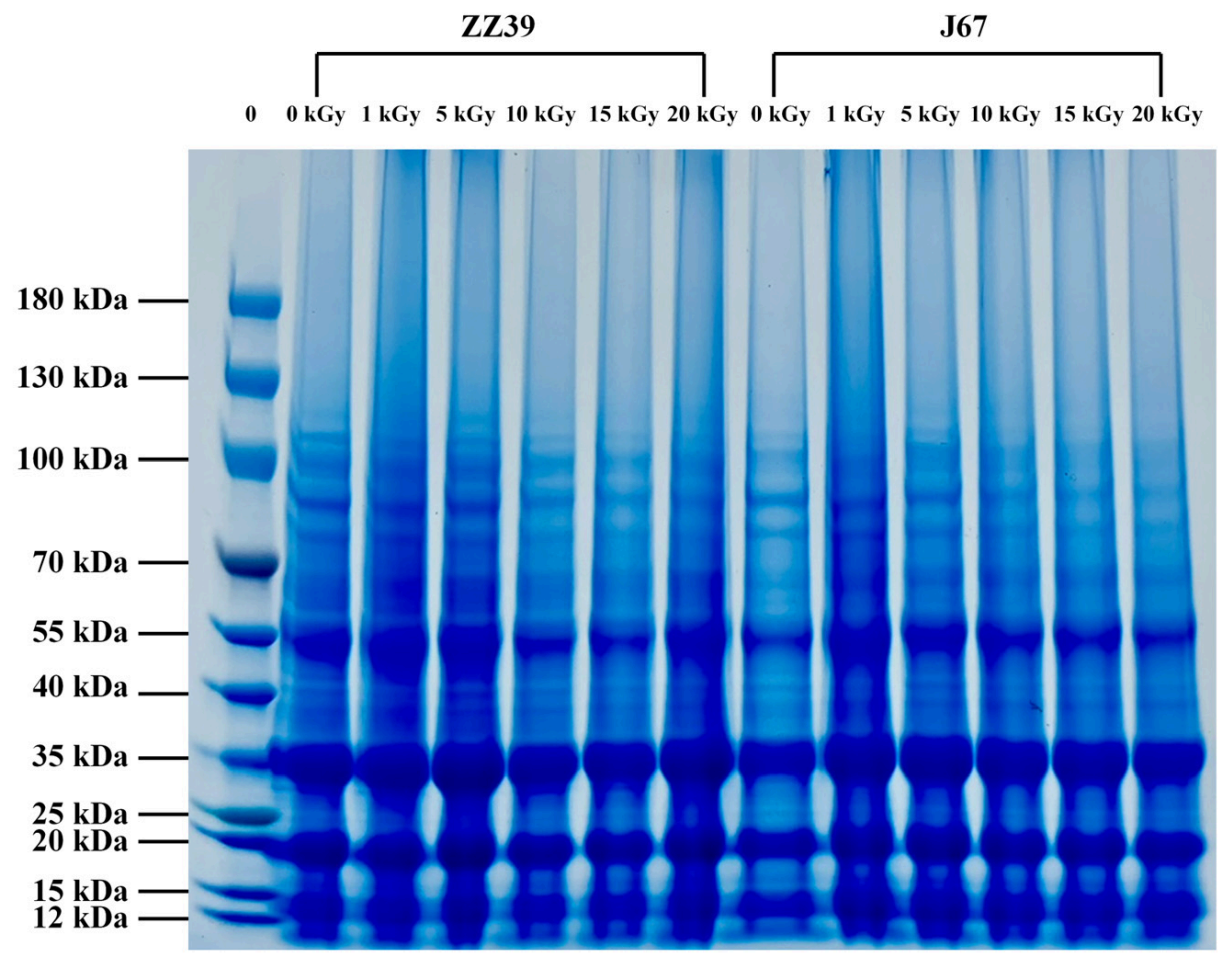
Reducing SDS-PAGE profiles of ZZ39 and J67 BRPIs following treatment with 0, 1, 5, 10, 15, or 20 kGy EBI. M, Vazyme MP201 molecular-weight marker (12–180 kDa); lane assignments correspond to the dose labels shown above the gel.

Reducing SDS-PAGE was used to examine the apparent molecular-weight distribution of the principal reducible protein subunits. Both BRPIs showed prominent bands at approximately 15, 20, 35, and 55 kDa (Figure 9). The major band positions were retained across 0–20 kGy, without a prominent new band or systematic migration shift. Differences in band intensity and smearing are treated as qualitative visual observations.

Li et al. observed retention of the principal band positions in oat and barley protein isolates after 0–20 kGy EBI, despite changes in band intensity [25]. Chickpea protein likewise showed no new reducing bands after 5–20 kGy EBI, although soluble-protein band intensity varied with dose [24]. At the wider range of 5–100 kGy, egg-white protein developed low-molecular-mass material at high doses [41], whereas this response was not observed in the present BRPIs at 0–20 kGy.

Retention of the principal band positions therefore indicates that EBI did not cause a gross redistribution of the major reducible subunits within the resolving range of the gel. It does not exclude oxidation, limited cleavage, or changes in noncovalent association that have little effect on electrophoretic mobility. Thus, the electrophoretic result shows the apparent molecular-weight profile under reducing conditions.

### 3.9. Effect of Electron Beam Irradiation on FTIR Spectra and Secondary Structure

**Figure 10:**
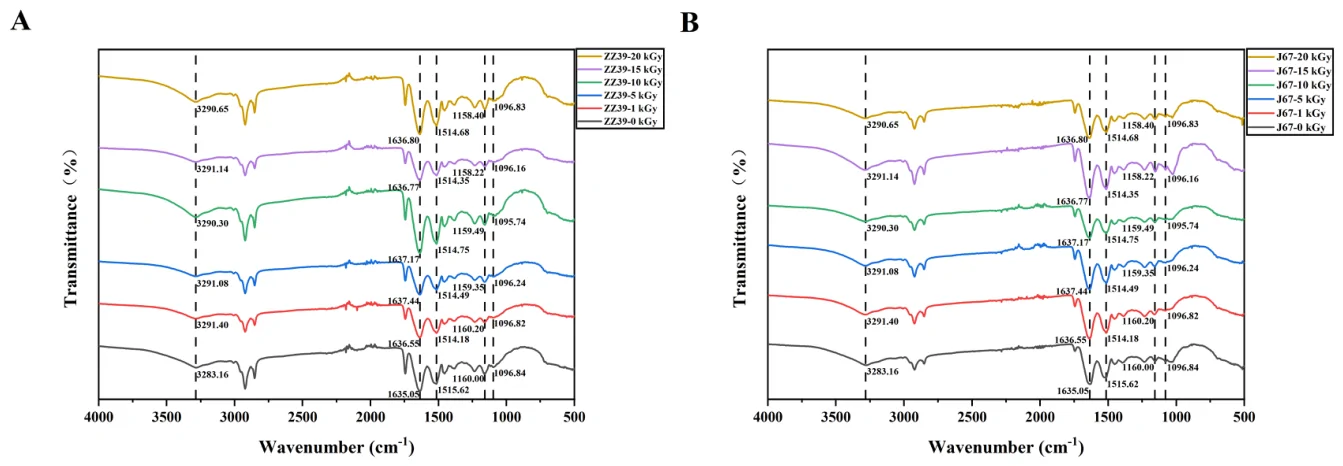
Fourier transform infrared spectra of (**A**) ZZ39 and (**B**) J67 BRPIs following treatment with different EBI doses.

FTIR spectroscopy was used to monitor protein-backbone vibrations. The overall spectral envelopes and major amide bands of both BRPIs were retained after EBI, without the appearance of a new dominant absorption band (Figure 10). These spectra indicate preservation of the principal protein vibrational features but do not exclude redistribution within the amide I region.

**Figure 11:**
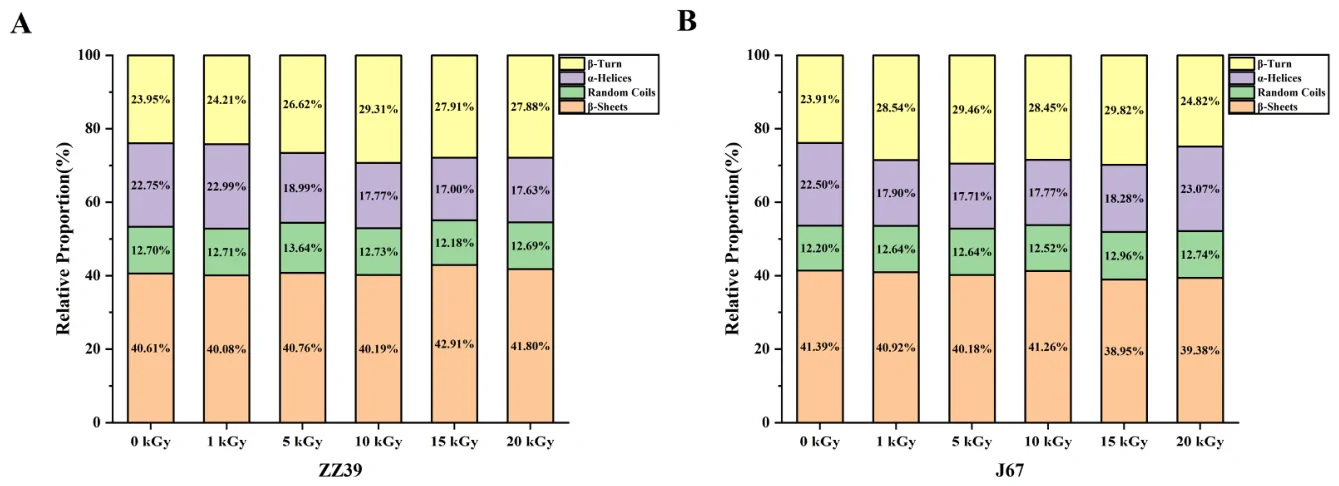
Relative secondary-structure proportions of (**A**) ZZ39 and (**B**) J67 BRPIs estimated by curve fitting of the amide I region.

Curve fitting of the amide I region provided estimates of relative secondary-structure proportions from the spectrum (Figure 11). In ZZ39, α-helix proportion was 22.75% in the control and remained similar at 1 kGy, but the values decreased at 5–15 kGy and increased slightly at 20 kGy. The β-turn proportion increased with irradiation doses up to 10 kGy, after which the values decreased, while the β-sheet and random-coil proportions changed within narrower ranges. In J67, α-helix proportion decreased at 1–15 kGy, and the β-turn proportion increased. However, when the irradiation dose increased to 20 kGy, the α-helix proportion increased to 23.07%, but the β-turn value decreased to 24.82%.

EBI-treated rice protein has similarly shown a decrease in α-helix content with increases in β-sheet, β-turn, and random-coil contents over 0–30 kGy [23]. Oat and barley protein isolates reached minimum α-helix content at 10 kGy followed by high-dose recovery [25]. In the present study, both BRPIs exhibited lower α-helix and higher β-turn proportions through 15 kGy. This redistribution persisted at 20 kGy in ZZ39 but returned close to the control proportions in J67. The fitted secondary-structure proportions differed across the available spectra. Therefore, EBI altered the fitted secondary-structure distribution in both BRPIs, with sustained differences in ZZ39 and a return toward control proportions at the highest dose in J67.

### 3.10. Effect of Electron Beam Irradiation on Intrinsic Fluorescence

**Figure 12:**
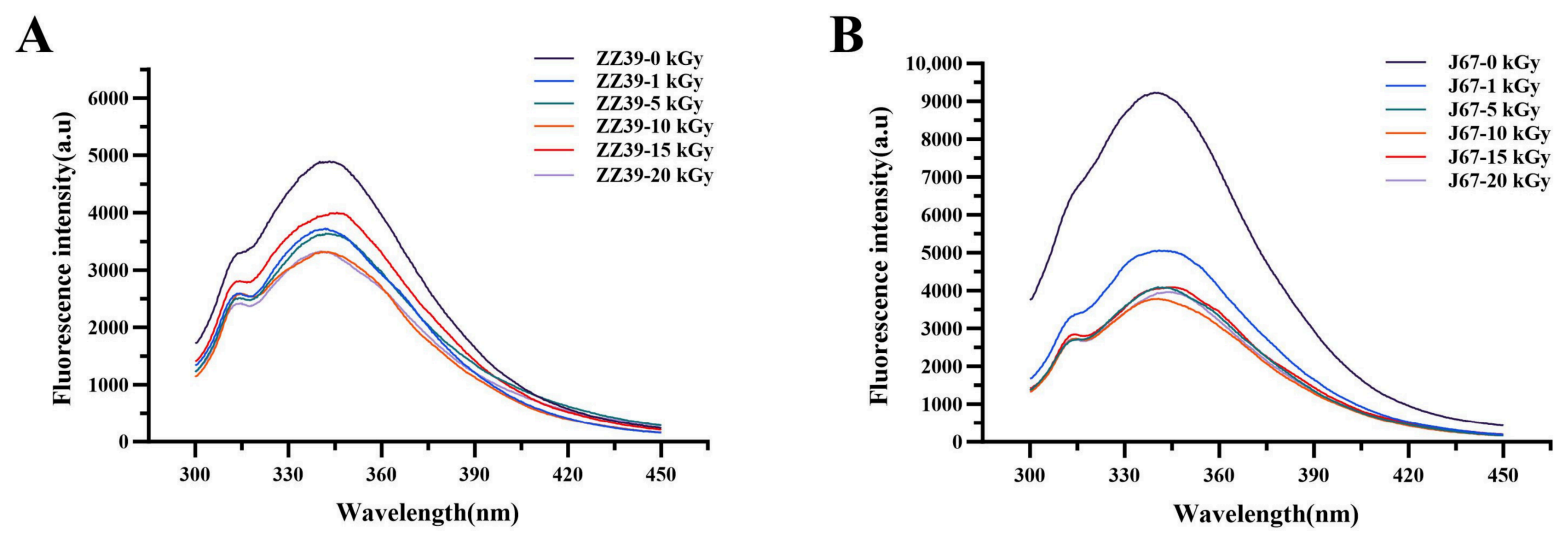
Intrinsic fluorescence spectra of (**A**) ZZ39 and (**B**) J67 BRPIs following treatment with different EBI doses.

Intrinsic fluorescence spectra were recorded to characterize the emission signal of the BRPI dispersions. EBI reduced fluorescence intensity in both BRPIs (Figure 12). For ZZ39, maximum intensity decreased from approximately 4888 in the control to 3720 at 1 kGy, recovered partially to 4239 at 5 kGy, and remained at approximately 3219–3504 at 10–20 kGy. For J67, it decreased from approximately 9437 to 4864 at 1 kGy and remained at approximately 3875–4026 at 5–20 kGy. The emission maximum varied only within 340.8–343.9 nm for ZZ39 and 341.5–343.5 nm for J67, without a systematic dose-dependent shift.

Li et al. similarly reported pronounced fluorescence quenching with only small changes in emission maximum for EBI-treated oat and barley protein isolates [25]. Protein states with altered tertiary packing can exhibit fluorescence changes without complete loss of secondary structure [42]. The present results therefore indicated that EBI altered the measured fluorescence intensity of both BRPIs, potentially reflecting changes in aromatic-residue environments as well as optical effects associated with particle-size differences.

### 3.11. Effect of Electron Beam Irradiation on Thermal Transitions

DSC was used to resolve the onset temperature (*T*_o_), peak transition temperature (*T*_d_), transition enthalpy (*ΔH*), and half-height peak width (*ΔT*_1/2_) of the thermal transition. In ZZ39, *T*_o_ increased from 75.80 °C to 81.95 °C at 10 kGy, decreased at 15 kGy, and increased again at 20 kGy. *T*_d_ showed an isolated increase from 127.53 to 137.32 °C at 1 kGy but remained at 129.97–131.15 °C from 5 to 20 kGy. *ΔH* decreased to 54.25–58.18 J/g at 1–5 kGy and increased to 71.93 and 69.97 J/g at 15 and 20 kGy, respectively; *ΔT*_1/2_ broadened at the two highest doses. In J67, *T*_o_ increased to 85.21–86.19 °C at 1–10 kGy, whereas *T*_d_ varied descriptively across the tested samples. *ΔH* decreased from 72.34 J/g to 65.42–65.88 J/g at 1–5 kGy, recovered at 10–15 kGy, and reached 80.94 J/g at 20 kGy; transition width was generally greater after irradiation (Table 2).

A closely related study reported the same decrease followed by recovery of *ΔH* in irradiated cereal proteins. Oat-protein *ΔH* decreased from 19.56 ± 0.71 to 17.64 ± 0.36 J/g at 10 kGy and recovered to 19.21 ± 0.32 J/g at 20 kGy, while barley protein changed from 22.66 ± 0.44 to 19.38 ± 0.46 and then 22.15 ± 0.35 J/g [25]. Cowpea protein generally showed lower thermal-transition enthalpy after irradiation [43], whereas sunflower protein showed progressive increases in both *T*_d_ and *ΔH* [44]. Such comparisons depend on sample hydration and the mass basis used for enthalpy. In the present BRPIs, reported half-height widths span approximately 56–64 °C, describing broad thermal events. Heterogeneous protein populations, overlapping transitions, and water-related contributions are possible explanations. The tabulated events are therefore described as thermal transitions rather than definitive measures of a single protein-denaturation process. The principal high-dose change was expressed through *ΔH* and broader transition peaks rather than a consistent shift in *T*_d_.

## 4. Conclusions

The present study examined brown rice quality and the properties of BRPIs recovered after protein isolation from dehulled paddy rice that had been treated with EBI. The findings therefore describe cultivar- and dose-associated differences in recovered proteins. J67 showed a descriptively greater color change than ZZ39, while both BRPIs showed variations across the tested dose levels in particle association and protein conformation, accompanied by changes in hydrophobicity, thiol/disulfide status, secondary and tertiary structure, and thermal behavior. No marked changes were observed in average surface potential or the major reducing SDS-PAGE band positions. No single dose produced favorable changes across all measured attributes. EBI conditions should therefore be selected according to cultivar and the intended quality target. Furthermore, the findings established physicochemical and structural changes of brown rice after irradiation, but the storage stability, as well as technological application-specific functionality remained to be further studied. Thus, subsequent studies should determine whether these structural changes improve storage stability or application-specific protein functionality.

## Figures and Tables

**Table 1:** CIELAB color coordinates *(L*^∗^, *a*^∗^, and *b*^∗^) and total color difference (*ΔE*) of ZZ39 and J67 brown rice following EBI treatment at 0–20 kGy.

Sample	*L* ^∗^	*a* ^∗^	*b* ^∗^	*ΔE*
ZZ39-0 kGy	53.49 ± 0.82 ^b^	3.02 ± 0.06 ^bc^	20.27 ± 0.49 ^a^	0.00
ZZ39-1 kGy	53.24 ± 0.06 ^b^	3.77 ± 0.01 ^a^	19.83 ± 0.13 ^a^	0.91
ZZ39-5 kGy	56.08 ± 0.94 ^a^	2.97 ± 0.05 ^bc^	20.60 ± 0.31 ^a^	2.61
ZZ39-10 kGy	51.16 ± 0.50 ^c^	2.86 ± 0.12 ^c^	20.38 ± 0.74 ^a^	2.33
ZZ39-15 kGy	50.99 ± 0.32 ^c^	1.49 ± 0.16 ^d^	20.60 ± 0.44 ^a^	2.95
ZZ39-20 kGy	50.21 ± 0.63 ^c^	3.25 ± 0.11 ^b^	19.78 ± 0.39 ^a^	3.32
J67-0 kGy	48.70 ± 0.20 ^a^	3.51 ± 0.17 ^ab^	17.46 ± 0.23 ^b^	0.00
J67-1 kGy	43.43 ± 0.01 ^b^	3.16 ± 0.01 ^bc^	16.07 ± 0.09 ^c^	5.46
J67-5 kGy	44.47 ± 0.66 ^b^	2.77 ± 0.09 ^c^	16.21 ± 0.15 ^c^	4.47
J67-10 kGy	42.81 ± 1.12 ^b^	3.41 ± 0.16 ^ab^	17.69 ± 0.28 ^b^	5.90
J67-15 kGy	44.43 ± 0.13 ^b^	3.81 ± 0.00 ^a^	17.40 ± 0.04 ^b^	4.28
J67-20 kGy	44.66 ± 0.86 ^b^	2.26 ± 0.20 ^d^	19.03 ± 0.17 ^a^	4.51

Values of *L*^∗^, *a*^∗^, and *b*^∗^ are presented as mean ± SD (*n* = 3). Within each rice material and column, different lowercase letters indicate significant differences among the available dose batches (*p* < 0.05).

**Table 2:** Differential scanning calorimetry parameters of ZZ39 and J67 BRPIs following treatment with different EBI doses.

Sample	*T*_o_ (°C)	*T*_d_ (°C)	*ΔH* (J/g BRPI)	*ΔT*_1/2_ (°C)
ZZ39-0 kGy	75.80 ± 0.93 ^c^	127.53 ± 0.28 ^b^	62.00 ± 0.27 ^b^	58.54 ± 1.08 ^b^
ZZ39-1 kGy	79.13 ± 0.48 ^abc^	137.32 ± 0.65 ^a^	58.18 ± 1.48 ^bc^	56.40 ± 1.77 ^b^
ZZ39-5 kGy	79.88 ± 0.57 ^abc^	130.65 ± 1.01 ^b^	54.25 ± 1.57 ^c^	58.49 ± 0.29 ^b^
ZZ39-10 kGy	81.95 ± 1.63 ^a^	129.97 ± 1.05 ^b^	60.42 ± 2.23 ^b^	59.17 ± 1.25 ^b^
ZZ39-15 kGy	77.21 ± 0.77 ^bc^	130.05 ± 0.16 ^b^	71.93 ± 1.31 ^a^	64.01 ± 0.61 ^a^
ZZ39-20 kGy	81.06 ± 2.16 ^ab^	131.15 ± 2.20 ^b^	69.97 ± 1.97 ^a^	64.16 ± 1.04 ^a^
J67-0 kGy	80.10 ± 0.43 ^b^	128.18 ± 0.49 ^a^	72.34 ± 0.44 ^b^	58.24 ± 0.71 ^b^
J67-1 kGy	86.19 ± 0.60 ^a^	130.06 ± 0.42 ^a^	65.42 ± 0.29 ^c^	60.77 ± 1.50 ^ab^
J67-5 kGy	86.05 ± 2.01 ^a^	131.35 ± 1.97 ^a^	65.88 ± 2.00 ^c^	61.88 ± 0.43 ^a^
J67-10 kGy	85.21 ± 1.32 ^a^	131.20 ± 2.12 ^a^	73.81 ± 1.10 ^b^	61.61 ± 1.06 ^ab^
J67-15 kGy	83.10 ± 1.41 ^ab^	132.00 ± 2.16 ^a^	73.74 ± 1.35 ^b^	61.97 ± 1.06 ^a^
J67-20 kGy	82.60 ± 1.51 ^ab^	129.45 ± 1.64 ^a^	80.94 ± 0.30 ^a^	61.60 ± 1.04 ^ab^

Values are presented as mean ± SD (*n* = 3). Within each rice material and parameter, different lowercase letters indicate significant differences among the available dose batches (*p* < 0.05). *T*_o_, onset temperature; *T*_d_, peak transition temperature; *ΔH*, transition enthalpy per gram of weighed BRPI powder; *ΔT*_1/2_, half-height width of the endothermic peak.

## Data Availability

The original contributions presented in the study are included in the article, further inquiries can be directed to the corresponding author.
